# Thymidylate synthase and methylenetetrahydrofolate reductase gene polymorphisms: relationships with 5-fluorouracil sensitivity

**DOI:** 10.1038/sj.bjc.6601523

**Published:** 2004-01-20

**Authors:** M-C Etienne, K Ilc, J-L Formento, P Laurent-Puig, P Formento, S Cheradame, J-L Fischel, G Milano

**Affiliations:** 1 Oncopharmacology Unit, Centre Antoine Lacassagne, 33 Avenue de Valombrose, 06 189 Nice Cedex 2, France; 2 Unité de Toxicologie Moléculaire, INSERM U490, 45 rue des Saints Pères, 75 270 Paris Cedex 06, France

**Keywords:** thymidylate synthase, methylenetetrahydrofolate reductase, pharmacogenetics, gene polymorphism, 5-fluorouracil, fluoropyrimidine

## Abstract

The relationship of thymidylate synthase (TS) and methylenetetrahydrofolate reductase (MTHFR) gene polymorphisms on 5-fluorouracil (FU) sensitivity was tested on 19 human cancer cell lines (head and neck, breast, digestive tract) in the absence and presence of folinic acid (FA) supplementation. Thymidylate synthase polymorphisms in the 5′ promoter region (double or triple tandem repeats) and 3′ untranslated region (6-bp deletion) were analysed by PCR. The C677T and A1298C MTHFR polymorphisms were determined by melting curve analyses (LightCycler). Thymidylate synthase activity and intracellular concentration of the reduced folate 5-10 methylenetetrahydrofolate (CH_2_FH_4_) were measured (biochemical assays). Thymidylate synthase activity was significantly different according to 5′ TS genotype, heterozygous cell lines (2R/3R) exhibiting higher TS activities than homozygous ones (*P*=0.05). However, whether in the absence or presence of FA, FU sensitivity was not statistically associated with either 5′ or 3′ TS polymorphism. Basal CH_2_FH_4_ cellular concentrations were lowest in C677T homozygous wild-type (wt) (C/C) cell lines. FU sensitivity was not linked to C677T polymorphism. In contrast, there was a marked trend for a greater FU efficacy in mutated A1298C variants (C/C+A/C) as compared to wt homozygous cell lines (A/A) (*P*=0.055 and 0.085 without and with FA supplementation, respectively). These results suggest for the first time a potential role of A1298C MTHFR polymorphism on fluoropyrimidine sensitivity.

5-Fluorouracil (FU) and fluoropyrimidine prodrugs remain the drugs of choice for the treatment of colorectal (Wils *et al*, 2001), breast (Fumoleau *et al*, 2003) and head and neck cancers (Posner *et al*, 2000). The conversion of FU into fluorodeoxyuridine monophosphate (FdUMP) leads to the inhibition of thymidylate synthase (TS, EC 2.1.1.45, the key enzyme of *de novo* deoxythymidine 5′-monophosphate synthesis, Figure 1

**Figure 1:**
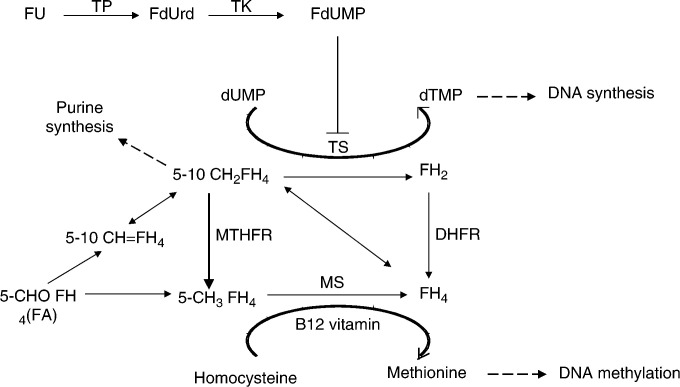
Folate metabolic pathways related to MTHFR and TS. 5-10 CH=FH_4_, 5-10 methenyltetrahydrofolate; 5-10 CH_2_FH_4_, 5-10 methylenetetrahydrofolate; 5-CH_3_FH_4_, 5-methyltetrahydrofolate; 5-CHOFH_4_ (FA), 5-formyltetrahydrofolate; DHFR, dihydrofolate reductase; dTMP, deoxythymidine 5′-monophosphate; dUMP, deoxyuridine 5′-monophosphate; FdUMP, 5-fluorodeoxyuridine 5′-monophosphate; FdUrd, 5-fluorodeoxyuridine; FH_2_, dihydrofolate; FH_4_, tetrahydrofolate; FU, 5-fluorouracil; MS, methionine synthase; MTHFR, 5-10 methylenetetrahydrofolate reductase; TK, thymidine kinase; TP, thymidine phosphorylase; TS, thymidylate synthase.

) and subsequent DNA synthesis arrest. The presence of FdUMP induces the formation of an inactive ternary complex between TS, FdUMP, and the methyl donor-reduced folate 5-10 methylenetetrahydrofolate (CH_2_FH_4_). Experimental studies have clearly established that the stabilisation of the ternary complex, and thus optimal TS inhibition, requires elevated cellular concentrations of CH_2_FH_4_ (Danenberg and Danenberg, 1978; Houghton *et al*, 1981; Rustum *et al*, 1987; Keyomarsi and Moran, 1988; Chéradame *et al*, 1997a). Accordingly, clinical studies have demonstrated higher antitumour efficacy when FU is associated with folinic acid (FA), a precursor of CH_2_FH_4_ (Petrelli *et al*, 1987; Poon *et al*, 1989; Doroshow *et al*, 1990; Piedbois *et al*, 1992; Jäger *et al*, 1996). We previously closely studied the role of FA supplementation and CH_2_FH_4_ intratumoral concentration on FU efficacy in a panel of 14 human cell lines (Chéradame *et al*, 1997a). Moreover, in patients receiving cisplatin–FU chemotherapy, we previously supplied direct evidence of the role of intratumoral CH_2_FH_4_ on FU efficacy, with significantly lower tumoral CH_2_FH_4_ concentrations in nonresponding patients as compared to responding patients (Chéradame *et al*, 1997b).

The intracellular CH_2_FH_4_ concentration is mainly controlled by the methylenetetrahydrofolate reductase enzyme (MTHFR, EC 1.5.1.20), which irreversibly converts CH_2_FH_4_ into 5-methyltetrahydrofolate (Figure 1) (Scott and Weir, 1994). Methylenetetrahydrofolate reductase is located at a major metabolic crossroad, directing the folate pool towards remethylation of homocysteine to methionine (and subsequent DNA methylation) at the expense of DNA synthesis (Figure 1). Methylenetetrahydrofolate reductase gene is located on chromosome 1p, and is subject to several polymorphisms (Rozen, 1996). Among them, the C677T (exon 4) and A1298C (exon 7) single-nucleotide polymorphisms (SNPs) are the two most commonly linked with altered phenotypes, both associated with lower enzyme activity (Frosst *et al*, 1995; Weisberg *et al*, 1998). The frequency of the mutated 677 TT genotype is around 10–15% in Caucasians, and only a few percent in Afro-Americans (Ueland *et al*, 2001). The 677C>T mutation enhances the thermolability of the enzyme (Frosst *et al*, 1995). The mutated 677 TT genotype is associated with elevated plasma homocysteine concentrations, as well as DNA hypomethylation that is involved in carcinogenesis processes. The influence of C677T polymorphism on disease risk is closely related to the nutritional folate status. In case of folate intake deficiency, 677 TT genotype is related to increased risk of congenital neural tube defect and to colorectal cancer (Ueland *et al*, 2001). The A1298C genotype has been less extensively studied (Van der Put *et al*, 1998; Weisberg *et al*, 1998) and the frequency of the mutated 1298 CC genotype reported in Japanese patients is around 3% (Urano *et al*, 2002). Interestingly, Urano *et al* (2002) recently reported that the mutated 677 TT genotype was associated with greater methotrexate toxicity, whereas the mutated 1298 CC genotype was linked with better methotrexate efficacy. Since MTHFR enzymatic deficiency may theoretically favour thymidine synthesis via an increase of CH_2_FH_4_, one can hypothesise that tumours exhibiting mutated MTHFR genotypes linked to enzymatic deficiency may be more sensitive to FU cytotoxicity than wild-type (wt) MTHFR genotype tumours. Such a possible impact of MTHFR genotype may also affect normal host tissues. So far, the influence of C677T and A1298C polymorphisms on fluoropyrimidine sensitivity and/or toxicity remains unknown.

Numerous experimental and clinical studies have previously shown that elevated tumoral TS activity or expression is related to FU resistance (Beck *et al*, 1994; Peters *et al*, 2002). The TS gene is located on chromosome 18p. A genetic polymorphism has been reported in the 5′ regulatory region (*cis*-acting enhancer element) of the TS promoter that presents either double or triple tandem repeats of a 28 bp sequence in Caucasian and Asian populations (Horie *et al*, 1995; Marsh *et al*, 1999). In an expression assay, Horie *et al* (1995) first reported that expression of the gene with triple repeat was higher than that of the gene with double repeat. Clinical studies have reported that triple repeat homozygous tumors (3R/3R) exhibit either higher TS mRNA or TS protein levels as compared to double repeat homozygous (2R/2R) (Kawakami *et al*, 2001b; Pullarkat *et al*, 2001). Recent clinical studies performed on small sets of patients have suggested that 5′ TS polymorphism may influence fluoropyrimidine sensitivity, with lower response rate in homozygous 3R/3R patients as compared to others (Marsh *et al*, 2001; Pullarkat *et al*, 2001; Villafranca *et al*, 2001; Park *et al*, 2002). We recently analysed 5′ TS genotype on a large set of metastatic colorectal cancer patients receiving FU-based therapy and found similar response rates in 3R/3R, 3R/2R and 2R/2R patients (Etienne *et al*, 2002). A second TS polymorphism consisting of a 6 bp deletion at bp 1494 in the 3′ untranslated region has recently been reported (Ulrich *et al*, 2000). The deleted 3′ variant is associated with decreased TS mRNA levels in colorectal tumours (Ulrich *et al*, 2000). No study has been reported on the relationship between 3′ TS polymorphism and fluoropyrimidine sensitivity.

The purpose of the present study was to analyse the relationship between major TS and MTHFR polymorphisms on FU sensitivity. To this end, we investigated a large panel of 19 human cancer cell lines representative of fluoropyrimidine-treated tumours (digestive, breast, head and neck), and expressing spontaneous sensitivity to FU. Special attention was paid to the reduced folate status, and FU sensitivity was evaluated in the absence or presence of FA supplementation in order to take into account the different fluoropyrimidine-based protocols administered to patients. In addition to the possible relationships between studied polymorphisms and FU cytotoxicity, we analysed the relationship between TS activity and polymorphisms, as well as links between CH_2_FH_4_ basal concentrations and MTHFR polymorphisms.

## MATERIAL AND METHODS

### Primers and probes

Primers and probes were all synthesised by Proligos (Paris, France). For TS polymorphisms, forward and reverse primers were GTGGCTCCTGCGTTTCCCCC and GCTCCGAGCCGGCCACAGGCA, respectively, for 5′ TS polymorphism, and GACGAATGCAGAACACTTCT and AATCTGAGGGAGCTGAGTAAC, respectively, for 3′ TS polymorphism.

For MTHFR polymorphisms, anchor probes were labelled on the 3′ extremity with fluorescein. Mutated specific probes were labelled at the 5′ extremity with LC-Red-640 for C677T and LC-Red-705 for A1298C variants and were phosphorylated on their 3′ extremity to avoid extension by PCR.

Primers for C677T variants were: *forward* 5′-TGG CAG GTT ACC CCA AAG G-3′; *reverse* 5′-TGA TGC CCA TGT CGG TGC-3′. Labelled probes for C677T variants were: *anchor* 5′-TGA GGC TGA CCT GAA GCA CTT GAA GGA GAA GGT GTC T-fluo; *C variant* (wild type) 5′-Red 640-CGG GAG **C**CG ATT TCA TCA T-3′ phos; *T variant* (mutated) 5′-Red 640-CGG GAG **T**CG ATT TCA TCA T-3′ phos. Primers for A1298C variants were: *forward* 5′-CTT TGG GGA GCT GAA GGA CTA CTA C-3′; *reverse* 5′-CAC TTT GTG ACC ATT CCG GTT TG-3′. Labelled probes for A1298C variants were: *anchor* 5′-AAG GAG GAG CTG CTG AAG ATG TGG GGG GAG GAG CT-fluo; *A variant* (wild type) 5′-Red 705-ACC AGT G**A**A GAA AGT GTC TTT GA-3′ phos; *C variant* (mutated) 5′-Red 705-ACC AGT G**C**A GAA AGT GTC TTT GA-3′ phos.

### Cell lines

This study was conducted on a panel of 19 human cancer cell lines (six breast, seven digestive tract, five head and neck, one pancreas) expressing spontaneous sensitivity to FU (not previously exposed to FU), with IC_50_ ranging from 0.3 to 25 *μ*M (Table 1

**Table 1:** Cell line characteristics and FU sensitivity (mean±s.e. from three separate experiments)

		**Cell lines**				
**Tumour type**		**Name**	**Origin**	**FU IC_50_ (*μ*M)**	**Opt FU IC_50_ (*μ*M)**	**Basal CH_2_FH_4_ (pmol mg^−1^ protein^−1^)**
	{	MCF7	Pr Rochefort	6.3±2.0	1.3±0.3	0.9±0.3
		T47D	Pr Rochefort	10.2±1.1	1.8±0.2	ND
		CAL51	CAL	4.1±0.1	4.1±0.1	ND
Breast		ZR75	Pr Rochefort	1.0±0.3	0.2±0.4	0.5±0.1
		CAL85-2	CAL	2.0±0.1	0.6±0.1	ND
		CAL120	CAL	7.0±1.3	2.1±0.2	ND
						
	{	CAL14	CAL	2.9±0.6	1.5±0.2	ND	
		WIDR	EORTC	3.6±0.2	0.9±0.1	0.4±0	
		COLO205	ATCC (CCL222)	0.8±0.1	0.2±0.1	3.3±2.1	
Colon		SW620	ATCC (CCL227)	13.8±2.7	7.3±0.3	0.4±0.1	
		SW403	ATCC (CCL230)	0.6±0.1	0.2±0.3	ND	
		CAL124	CAL	0.3±0.1	0.1±0.4	ND	
						
Intestine		HUTU80	ATCC (HTB40)	9.7±0.9	9.7±0.1	ND	
							
Pancreas		HS766T	ATCC (HTB134)	16.5±2.4	8.3±0.3	ND	
	{	CAL33	CAL	0.6±0.1	0.2±0.2	ND	
		CAL27	CAL	1.8±0.2	0.4±0.3	0.8±0.3	
Head and neck		Hep2	ATCC (CCL23)	25.4±3.5	8.6±0.1	ND	
		KB	ATCC (CCL17)	7.3±1.1	1.6±0.2	1.3±0.1	
		Detroit 562	ATCC (CCL138)	2.9±1.0	1.0±0.3	0.3±0	

Opt FU IC_50_=optimal FU IC_50_ obtained with FA supplementation (see Material and Methods); ND=not detectable; CH_2_FH_4_=methylenetetrahydrofolate. Cell line origins: CAL cell lines come from our institute; Pr Rochefort is from INSERM U 184, Montpellier, France; EORTC, European Organisation for Research and Treatment of Cancer; ATCC, American Type Culture Collection (Rockeville, MD, USA).

). Cell doubling time ranged from 1.3 to 6.3 days (mean 2.7, median 2.2).

### Biochemical investigations

Of the 19 cell line panel, 14 had been previously investigated for FU sensitivity with or without FA supplementation, intracellular reduced folate content, and TS activity (Chéradame *et al*, 1997a), as briefly described below. In order to match the physiological circulating folate concentration in humans, cells were grown in a folate-controlled medium for 10 days before experiments were started (folate-free DMEM medium supplemented with 40 nM of *dl-*5-methyltetrahydrofolate, 0.1 mM of *l*- ascorbic acid for folate stabilisation, 10% FBS, 2 mM glutamine, 50 000 U l^−1^ penicillin and 80 *μ*M streptomycin) and all experiments have been subsequently performed in this folate-controlled medium.

#### Cytotoxicity experiments

Cells were exposed for 5 days to various FU (14 concentrations ranging from 0.01 to 500 *μ*M), FA (6 concentrations ranging from 0.01 to 300 *μ*M of pure *l*-FA) or FU+FA concentrations (sextuplicates in 96-well microtitration plates). Growth inhibition was assessed by the MTT test (Carmichael *et al*, 1987) and the dose–effect curves were analysed on GraphPad software (ISI, USA).

#### Intracellular reduced folate measurement

After 5days of growth (175 cm^2^ plates), cells were harvested, washed three times in phosphate buffer saline at +4°C and cell pellets containing approximately 50 × 10^6^ cells were stored in liquid nitrogen. 5-10 Methylenetetrahydrofolate was measured on a 15 000 **g** cytosol, as previously described (Chéradame *et al*, 1997a), based on the entrapment assay initially developed by Bunni *et al* (1988). This assay is based on the stoicheiometric formation of a stable ternary complex between CH_2_FH_4_, excess purified TS (0.225 *μ*M final concentration), and excess ^3^H-FdUMP (0.35 *μ*M final concentration). Recovery calculated from controls containing known CH_2_FH_4_ concentrations was 90% on average. Sensitivity limit was 0.3 pmol mg^−1^ protein. Intra- and interassay reproducibility were 9.4 and 25.0%, respectively.

#### Measurement of TS activity

Thymidylate synthase activity was measured according to the tritium-release assay initially described by Spears and Gustavsson (1988) and modified by us (Etienne *et al*, 2002). Cells (4 × 10^6^ cells ml^−1^ in 50 mM Tris HCl buffer pH 7.3 containing 2 mM dithiothreitol) were sonicated on ice bed (three times at 10 s intervals) and centrifuged at 100 000 **g** for 30 min (+4°C). The assay consisted in incubating 25 *μ*l of cytosol with excess ^3^H-dUMP (1 *μ*M final concentration) and CH_2_FH_4_ (0.62 mM final concentration) in a total volume of 55 *μ*l (in the previous buffer). After 0, 10, 20 and 30 min of incubation at +37°C, the reaction was stopped on ice bed. The excess of ^3^H-dUMP was removed by adding 300 *μ*l of activated charcoal (15%) containing 4% trichloracetic acid (5 min centrifugation at 14 000 **g**, room temperature). The ^3^H_2_O formed during the incubation was then measured in an aliquot of the above supernatant. Results were expressed as fmoles of ^3^H_2_O formed per min per mg of protein, based on the linear regression obtained from the incubation times. Cytosolic proteins were determined by the Bradford colorimetric assay (Protein Assay Reagent, Biorad Laboratories, Germany) with human serum albumin as standard. The sensitivity limit was 10 fmol min^−1^ mg^−1^ protein. The CV for interassay reproducibility (*N*=7) was 15%.

### Gene polymorphisms

Thymidylate synthase and MTHFR genetic polymorphisms were analysed on DNA extracted from cell pellets stored in liquid nitrogen.

#### Determination of 5′ and 3′ TS polymorphisms

For the 5′ polymorphism, a fragment containing the 28-bp repeats was amplified (expected fragment size was 220 bp for 2R and 248 bp for 3R). For the 3′ polymorphism, a fragment containing the 6 bp deletion was amplified (expected fragment sizes were 110 bp for the wild type and 104 bp for the variant allele). In each case, PCR were run on a GeneAmp^R^ PCR system 9700 (Applied Biosystems, Courtabœuf, France) in a 25 *μ*l final volume containing 50 ng of genomic DNA, 1 mM MgCl_2_, 2.5 *μ*l of buffer 10 ×, 1.25 mM of dNTPs, 0.15 *μ*M of each specific forward and reverse primer and 0.05 U *μ*l^−1^ of *Taq* polymerase Cetus (Perkin Elmer, Courtaboeuf, France). After 30 cycles of amplification (denaturation at 94°C for 30 s, annealing at 62°C for 60 s, and extension at 72°C for 90 s), amplification products were electrophoresed on acrylamide gel at 8%. For 5′ polymorphism, products of 220 bp (2R/2R), 248 bp (3R/3R) or both (2R/3R) were observed. For 3′ polymorphism, products of 110 bp (6 bp/6 bp), 104 bp (0 bp/0 bp) or both (6 bp/0 bp) were observed.

#### Determination of MTHFR polymorphisms

**Figure 2:**
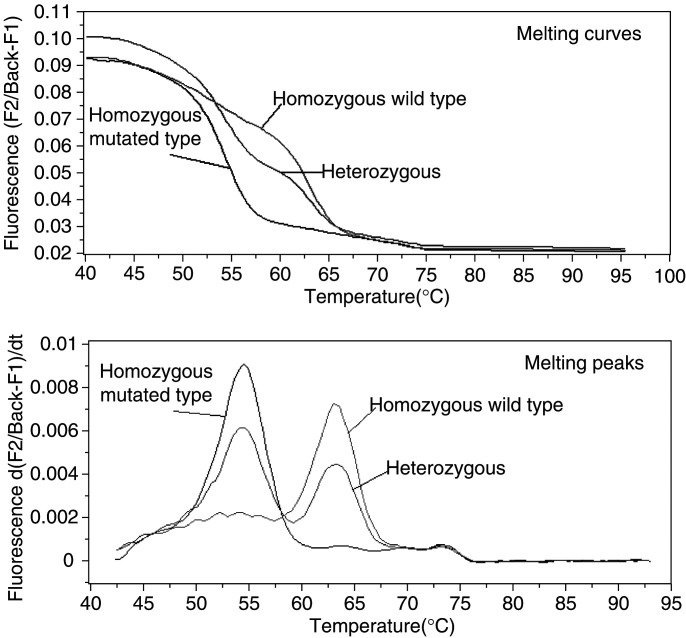
Typical example of melting curves and melting peaks used to genotype C677T MTHFR gene mutation. A wt probe is used. Melting peak temperatures obtained from a derivative of the melting curves are 64°C for homozygous wt (CC), 64°C and 55°C for heterozygous (CT) and 55°C for homozygous mut types (TT).

The C677T (Ala → Val) and A1298C (Glu → Ala) variants were analysed simultaneously by means of melting curve analyses on LightCycler (Roche), based on the fluorescence resonance energy transfer (FRET) principle (see Figure 2

for a typical example of analysis). We used a method derived from that initially described by Nakamura *et al* (2002). A duplex PCR amplification was first run in 20 *μ*l final volume containing 80 ng genomic DNA (2 *μ*l), 2 *μ*l of ready-to-use Hotstart PCR mixture (LightCycler Faststart DNA Master Hybridization Probes kit, Roche Diagnostic, France), 3 mM MgCl_2_, 0.2 *μ*M of each primer, 0.2 *μ*M of each specific anchor fluorescein-labelled probe, and 0.4 *μ*M of each specific wt variant (C 677 or A 1298 LC-red-labelled probe), in PCR grade water. After DNA denaturation and enzyme activation (8 min at 95°C), DNA was amplified for 45 cycles (15 s at 95°C, 10 s at 55°C and 10 s at 72°C). At the end of PCR, melting curves of the DNA/probes complexes synthesised were performed by increasing temperature gradually (0.1°C s^−1^) up to 95°C. Methylenetetrahydrofolate reductase variant identification was based on the fact that the melting temperature of the DNA/probe complex is lower in the case of DNA/probe T/C mismatch at nucleotide 677 or DNA/probe C/A mismatch at nucleotide 1298. The C677T genotype was monitored at 640 nm and the melting curve showed a single peak at 64°C for C/C samples, a single peak at 55°C for T/T samples, and two peaks for heterozygous cell lines. The A1298C genotype was monitored at 705 nm and the melting curve showed a single peak at 63°C for A/A samples, a single peak at 60°C for C/C samples, and two peaks for heterozygous cell lines. In the event of DNA/probe mismatch, a second PCR (duplex PCR if necessary) was conducted with specific mutated (mut) variants (T 677 or C 1298 LC-red-labelled probes), in order to confirm the presence of the specific analysed mutation.

### Data analysis and statistics

All experiments were performed in triplicate. 5-Fluorouracil IC_50_ was defined as the concentration causing 50% growth inhibition as compared to control cells. In the conditions with FA supplementation, for each tested FA concentration we computed a potentiation factor (*F*) equal to the FU IC_50_ without FA divided by the FU IC_50_ with FA. Optimal FU IC_50_ corresponded to the IC_50_ obtained in the presence of optimal FA, that is, allowing 90% of the maximal *F* value to be reached. Correlations between TS activity and FU IC_50s_ were tested by means of Pearson correlation, after logarithm transformation that allows the Gaussian distribution to be fitted. All other statistics were performed by means of nonparametric tests: Spearman rank correlation, Kruskal–Wallis test, Mann–Whitney test. For this latter test, mut *vs* wt comparison was performed by merging homozygous and heterozygous mutated variants *vs* homozygous wt cell lines. Statistics were performed on SPSS software (Chicago, USA).

## RESULTS

### Cell line sensitivity to FU±FA, intracellular reduced folates and TS activity

Table 1 summarises cell line sensitivity to FU alone or in combination with optimal FA concentration. In two cell lines (CAL51 and HUTU 80), FU cytotoxicity was not enhanced by FA. The basal intracellular CH_2_FH_4_ concentration was detectable in nine cell lines out of the 19 investigated. Thymidylate synthase activity (Table 2

**Table 2:** Description of TS activity, TS and MTHF polymorphisms

**Cell lines**				**TS polymorphisms**		**MTHFR polymorphisms**	
**Tumour type**		**Name**	TS activity^*^ (pmol min^−1^ mg^−1^ protein)	**5′ genotype**	**3′ genotype**	**C677T genotype**	**A1298C genotype**
	{	MCF7	18.1±3.0	3R/3R	6 bp/6 bp	T/T	A/A
		T47D	29.1**±**1.0	2R/2R	6** **bp/6 bp	T/T	A/A
		CAL51	48.6±5.6	2R/3R	6 bp/6 bp	C/T	A/C
Breast		ZR75	14.4±0.9	2R/3R	6 bp/6 bp	C/T	A/C
		CAL85-2	41.6±0.9	2R/3R	6 bp/0 bp	C/C	A/A
		CAL120	17.1±1.5	3R/3R	6 bp/0 bp	C/C	A/A
							
	{	CAL14	11.6±2.1	2R/2R	6 bp/6 bp	C/T	A/C	
		WIDR	9.5±3.0	2R/2R	6 bp/6 bp	C/T	A/C	
		COLO205	19.9±3.8	2R/2R	6 bp/6 bp	C/T	A/A	
Colon		SW620	24.6±2.4	2R/2R	6 bp/6 bp	T/T	A/A	
		SW403	6.7±0.2	3R/3R	0 bp/0 bp	C/T	A/A	
		CAL124	7.2	2R/2R	6 bp/6 bp	C/C	C/C	
							
Intestine		HUTU80	82.8±10.0	2R/2R	6 bp/0 bp	C/T	A/A	
								
Pancreas		HS766T	17.2±0.3	3R/3R	6 bp/6 bp	C/C	A/A	
	{	CAL33	34.3±0.9	2R/3R	6 bp/6 bp	C/C	A/C	
		CAL27	38.4±5.8	2R/3R	6 bp/0 bp	C/C	C/C	
Head and neck		Hep2	58.3±3.9	2R/3R	0 bp/0 bp	C/T	A/A	
		KB	9.7±1.9	3R/3R	0 bp/0 bp	C/T	A/C	
		Detroit 562	18.6	3R/3R	0 bp/0 bp	C/T	A/C	

TS=thymidylate synthase; MTHFR=methylenetetrahydrofolate reductase.

) ranged between 6.7 and 82.8 pmol min^−1^ mg^−1^ protein (mean 27). The greater the FU sensitivity, the lower the TS activity (*P*=0.078 and 0.032, in the absence and presence of optimal FA concentration, respectively). Basal CH_2_FH_4_ was not linked to FU sensitivity.

### 5′ and 3′ TS polymorphisms

Distribution of 5′ TS genotype (Table 2) was 36.8% 2R/2R (*n*=7), 31.6% 2R/3R (*n*=6) and 31.6% 3R/3R (*n*=6). Distribution of 3′ TS genotype (Table 2) was 57.9% 6 bp/6 bp (*n*=11), 21.1% 6 bp/0 bp (*n*=4) and 21.1% 0 bp/0 bp (*n*=4). Cell doubling time was not linked to 5′ or 3′ TS genotypes.

**Figure 3:**
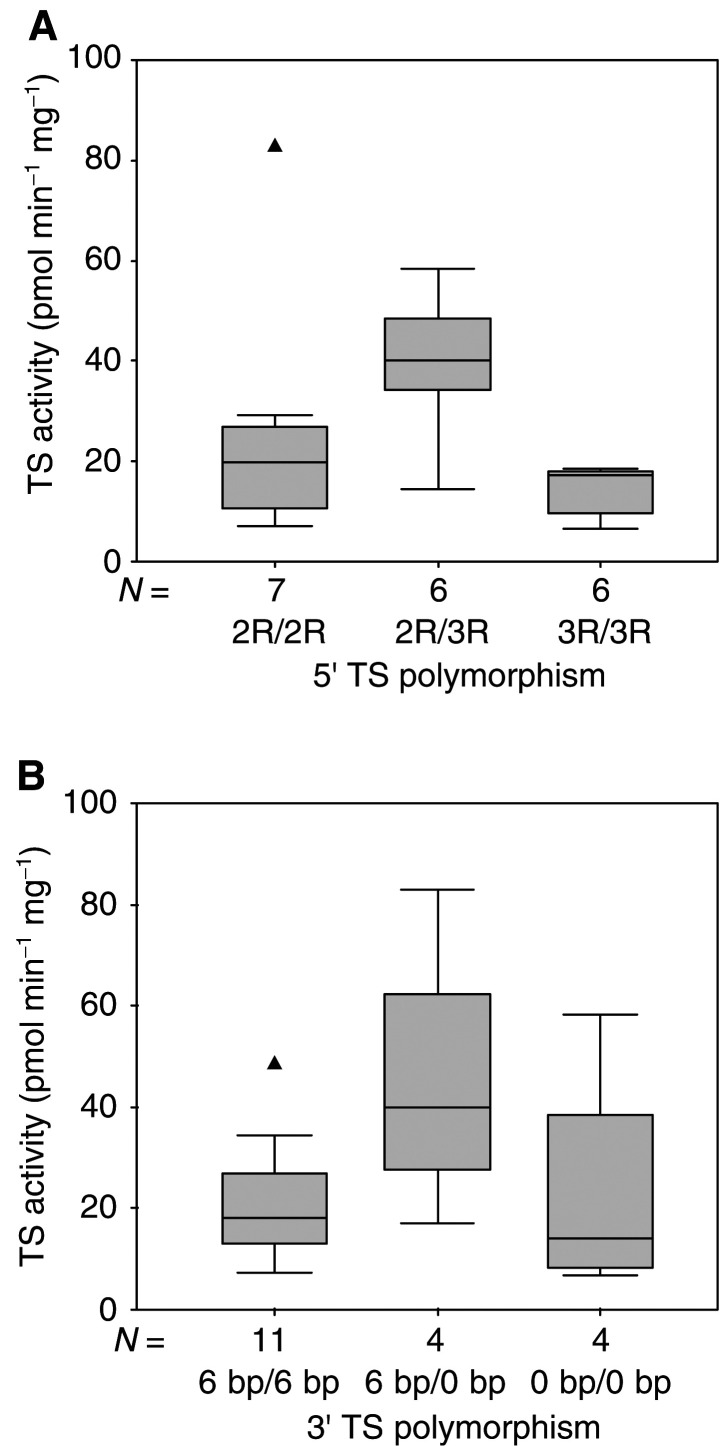
Distribution of TS enzymatic activity according to 5′ TS polymorphism (**A**) or 3′ TS polymorphism (**B**). Boxes delimit the first and third quartiles, with the median inside, and bars represent the range of values that fall within 1.5-fold the interquartile range. Triangles (▴) represent outliers, defined as individual values greater than 1.5-fold the interquartile range. Kruskall–Wallis *P*-values were 0.050 and 0.23 for (**A**) and (**B**), respectively.

**Figure 4:**
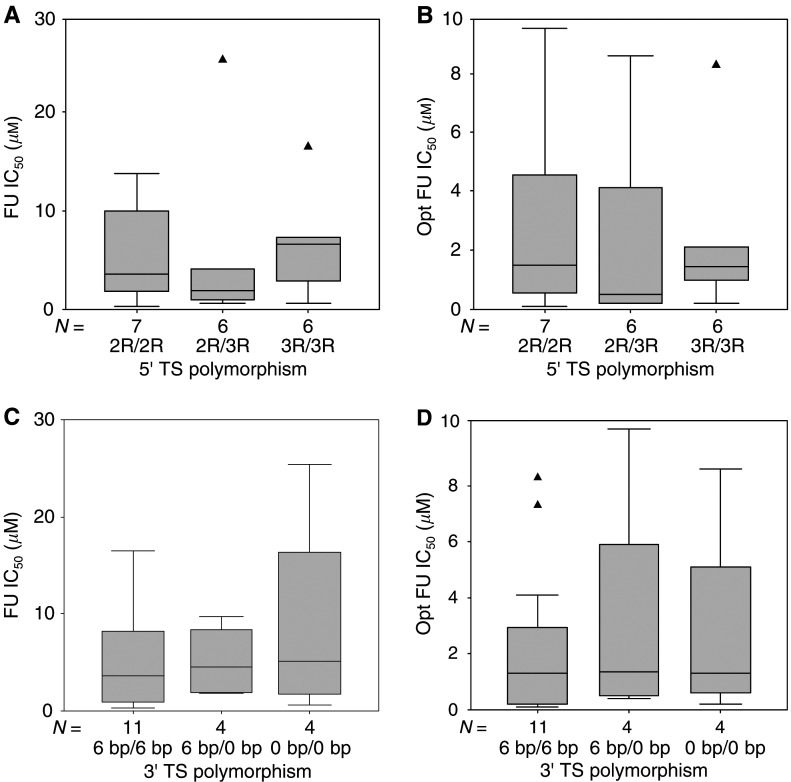
Distribution of FU sensitivity (FU IC_50_ in ‘physiological-folate’ condition, and Opt FU IC_50_ in the presence of optimal FA concentration) according to 5′ TS polymorphism (**A**, **B**) or 3′ TS polymorphism (**C**, **D**). Boxes delimit the first and third quartiles, with the median inside, and bars represent the range of values that fall within 1.5-fold the interquartile range. Triangles (▴) represent outliers, defined as individual values greater than 1.5-fold the interquartile range. Kruskall–Wallis *P*-values were 0.66, 0.81, 0.90 and 0.76 for (**A**), (**B**), (**C**) and (**D**), respectively.

Thymidylate synthase activity was significantly different according to 5′ TS genotype, heterozygous cell lines exhibiting significantly higher TS activities than homozygous ones (median 20, 40 and 17 pmol mn^−1^ mg^−1^ in 2R/2R, 2R/3R and 3R/3R, respectively; Kruskal–Wallis *P*=0.050, Figure 3A

). No significant relationship was observed between TS activity and 3′ TS genotype (Kruskal–Wallis *P*=0.23, Figure 3B). Whether in the absence or presence of FA, FU sensitivity was not statistically associated with either 5′ or 3′ TS polymorphism (Figure 4

).

### C677T and A1298C MTHFR polymorphisms

Distribution of C677T genotype was 31.6% C/C (*n*=6), 52.6% C/T (*n*=10), 15.8% T/T (*n*=3) and that of A1298C was 52.6% A/A (*n*=10), 36.8% A/C (*n*=7) and 10.5% C/C (*n*=2) (Table 2).

**Figure 5:**
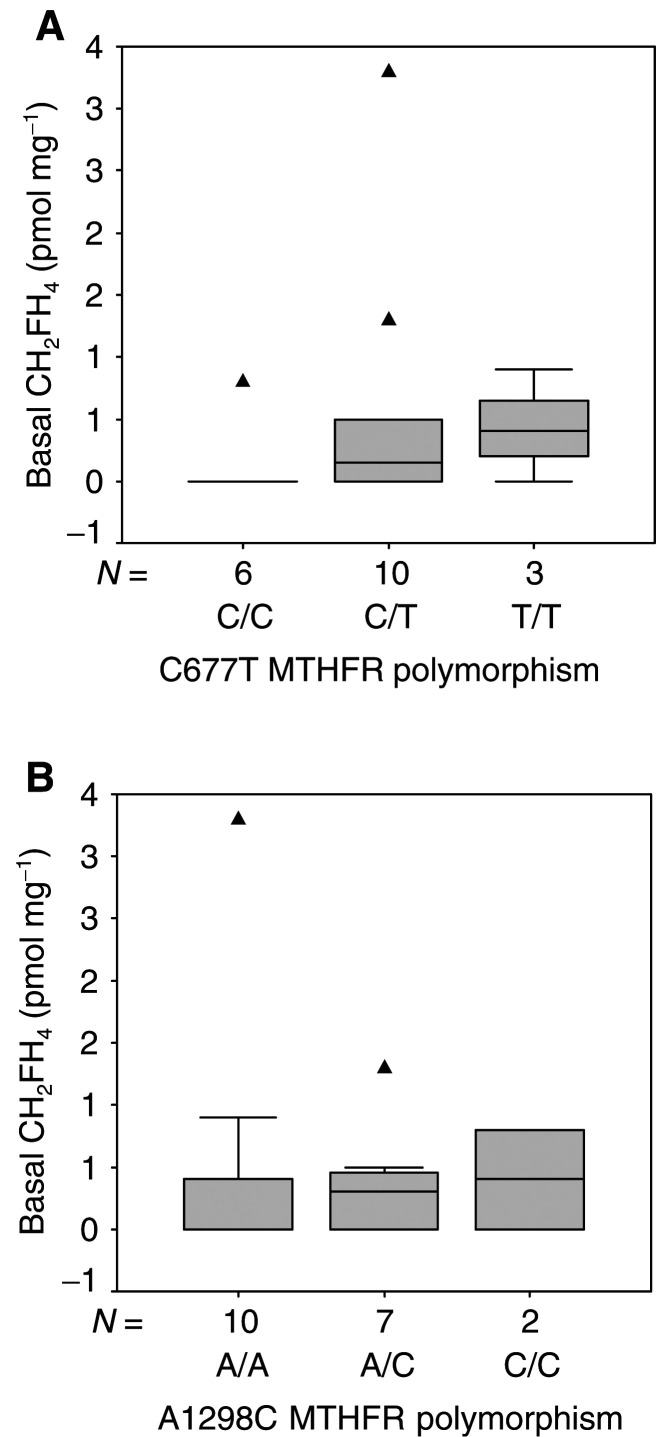
Distribution of basal CH_2_FH_4_ concentration according to C677T MTHFR polymorphism (**A**) or A1298C MTHFR polymorphism (**B**). Boxes delimit the first and third quartiles, with the median inside, and bars represent the range of values that fall within 1.5-fold the interquartile range. Triangles (▴) represent outliers, defined as individual values greater than 1.5-fold the interquartile range. Kruskall–Wallis *P*-values were 0.36 and 0.74 for (**A**) and (**B**), respectively.

From Tables 1 and 2, it appears that basal CH_2_FH_4_ concentrations were not detectable in 5/6 homozygous wt (C/C) 677 genotype, whereas detectable concentrations were observed in 7/13 mut (T/T and C/T) 677 genotype. Also, basal CH_2_FH_4_ concentrations were below detection limit in 7/10 homozygous wt (A/A) 1298 genotype, in contrast with detectable concentrations observed in 5/9 mut (C/C and A/C) 1298 genotype. The above observations, which did not reach statistical significance, are depicted in Figure 5

. Of note, in the three cell lines with homozygous wt genotype for both 677 and 1298 (CAL85-2, CAL120, HS766T), CH_2_FH_4_ was always below the detection limit.

**Figure 6:**
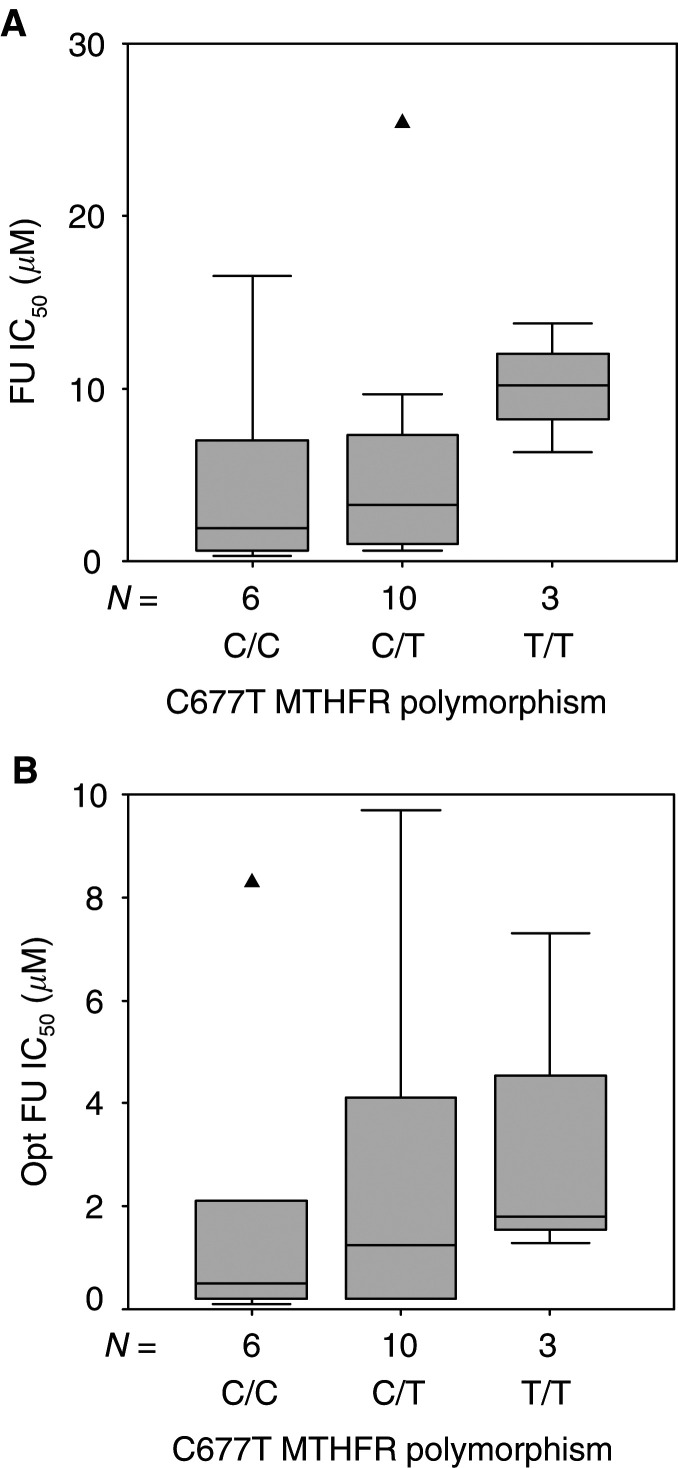
Distribution of FU IC_50_ (‘physiological-folate’ condition (**A**)) and Opt FU IC_50_ (in the presence of optimal FA concentration (**B**)) according to C677T MTHFR polymorphism. Boxes delimit the first and third quartiles, with the median inside, and bars represent the range of values that fall within 1.5-fold the interquartile range. Triangles (▴) represent outliers, defined as individual values greater than 1.5-fold the interquartile range. Kruskall–Wallis *P*-values were 0.20 and 0.45 for (**A**) and (**B**), respectively.

**Figure 7:**
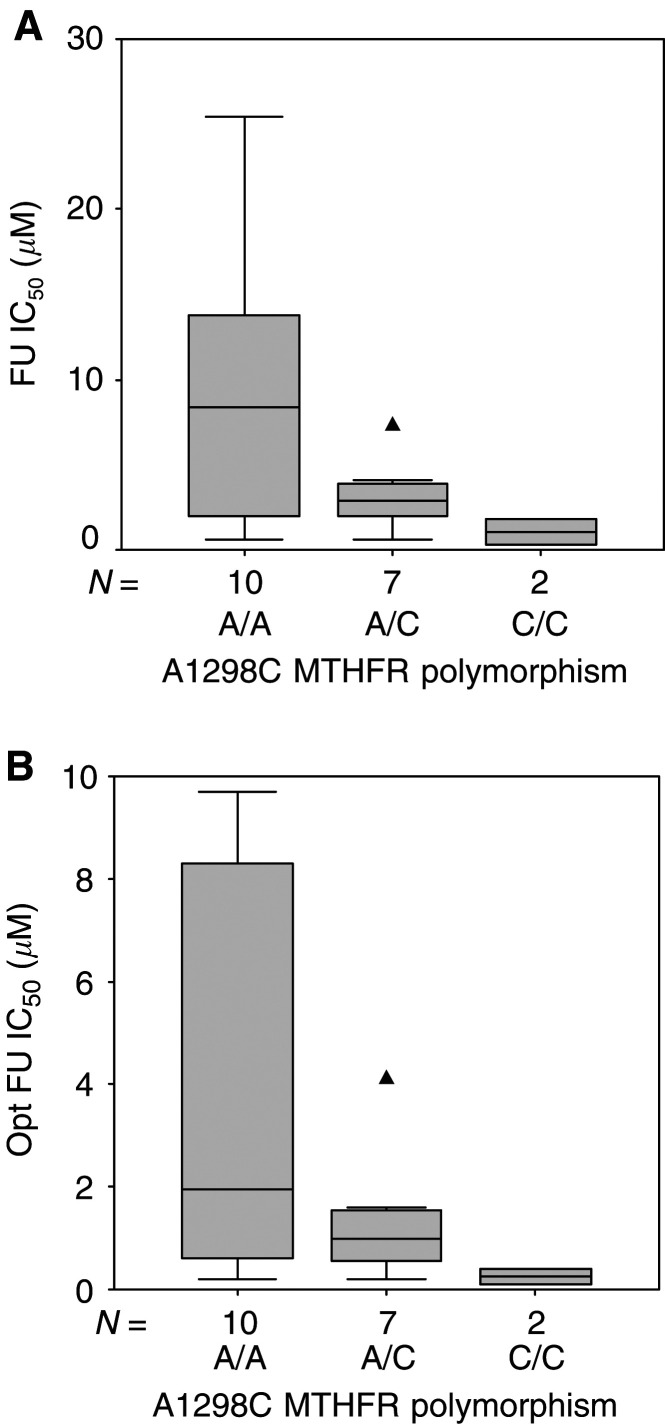
Distribution of FU IC_50_ (‘physiological-folate’ condition (**A**)) and Opt FU IC_50_ (in the presence of optimal FA concentration (**B**) according to A1298C MTHFR polymorphism. Boxes delimit the first and third quartiles, with the median inside, and bars represent the range of values that fall within 1.5-fold the interquartile range. Triangles (▴) represent outliers, defined as individual values greater than 1.5-fold the interquartile range. Kruskall–Wallis *P*-values were 0.086 and 0.11 for (**A**) and (**B**), respectively.

Whether in the absence or presence of FA, FU sensitivity (FU IC_50_ or optimal (Opt) FU IC_50_) was not linked to C677T genotype (C/C *vs* C/T *vs* T/T: nonsignificant, Figure 6

; wt (C/C) *vs* mut (C/T+T/T): nonsignificant). In contrast, FU efficacy tended to be higher in mutated A1298C variants (C/C+A/C), both in ‘physiological-folate’ conditions (Figure 7A

) and in the presence of optimal FA concentration (Figure 7B). In ‘physiological-folate’ conditions, FU IC_50_ ranged from 0.6 to 25.4 *μ*M (median 8.4) in the 10 cell lines exhibiting wt A/A 1298 variant, whereas IC_50_ were comprised between 0.3 and 7.3 (median 2.9) in the nine cell lines exhibiting mutated A/C or C/C 1298 variants; this difference was very close to statistical significance (Mann–Whitney, *P*=0.055). A similar pattern of distribution, although less significant (Mann–Whitney, *P*=0.085), was observed for Opt FU IC_50_, that is in the presence of optimal FA concentrations.

## DISCUSSION

In the context of cancer treatment, pharmacogenetic exploration may result, in the future, in the replacement of tedious and heavy phenotypic explorations either at blood level (pharmacokinetics) or at tumour level (prognostic/predictive markers) by genetic analyses performed on easily obtainable DNA samples from normal cells (blood or oral cavity brushing for instance). The observation that pharmacogenetic status faithfully reflects phenotypic changes at the target level is a prerequisite of clinical pharmacogenetic applications. In fact, from a theoretical point of view, genetic polymorphisms are identical in all tissues. However, the possibility of a clonal selection emerging from a heterozygous subject during the process of carcinogenesis cannot be ruled out. This strengthens the need to examine the impact of cancer-treatment-related gene polymorphisms at the tumoral target itself.

The aim of the present study was thus to analyse the impact at tumoral target level of polymorphisms of two major genes related to DNA synthesis, namely TS and MTHFR polymorphisms, which may influence FU cytotoxicity. To our knowledge, the present experimental study is the first one designed for this purpose. Moreover, the present model was specially controlled for reduced folate status (folate-free medium supplemented with physiological-compatible CH_2_FH_4_ concentrations) and experiments were conducted both in the absence and presence of optimal FA concentrations, in order to mimic the two opposite situations regarding FA supplementation in FU-based treated patients. To this end, we closely explored a panel of 19 cancer cell lines expressing spontaneous FU sensitivity and covering the major fluoropyrimidine-treated localisations (digestive tract, breast, head and neck). The 5′ TS and C677T MTHFR genotype frequencies were in the range of those reported in Caucasian populations (Marsh *et al*, 1999; Ueland *et al*, 2001), strengthening the relevance of the present model. The 3′TS and A1298C MTHFR polymorphisms have been less extensively studied in Caucasian populations, thus frequency comparisons with published data were difficult to perform. The chosen experimental approach did not allow allelotype analyses to be performed, since such analyses require large population studies.

Up to now, clinical studies investigating the influence of 5′ TS gene polymorphism on TS mRNA or protein level have given rather contrasting results. A retrospective study conducted on 52 colorectal tumour specimens reported that triple repeat homozygous (3R/3R) exhibit 3.6-fold higher TS mRNA levels as compared to double repeat homozygous (2R/2R) (Pullarkat *et al*, 2001). Another study conducted on 133 cancer biopsies (mostly colorectal cancer) showed no difference in TS mRNA level according to 5′ TS genotype, but demonstrated higher TS protein concentration in 3R/3R as compared to 2R/2R (Kawakami *et al*, 2001b). One of the major findings of the present study is that TS enzymatic activity is significantly influenced by the 5′TS genotype. This result is somewhat surprising since TS activity was significantly higher in 2R/3R heterozygous cell lines (Table 2, Figure 3A). However, superimposable conclusions were drawn from the only available data on the link between TS activity and TS polymorphism, which we recently published on colorectal cancer patients (Etienne *et al*, 2002). Mandola *et al* (2003) recently described an additional G → C SNP within the second repeat of the triple tandem that may influence the transcriptional activity of the gene. Such an additional polymorphism in the 5′ regulatory region with functional consequences on transcriptional activity may complicate the links between TS activity and tandem repeat polymorphisms. This could explain the present unexpected data with a high TS activity in 2R/3R cell lines. In contrast to 5′ TS genotype, the present experimental data do not support a significant influence of 3′ TS genotype on TS enzymatic activity.

Importantly, the present experimental model was powerful enough to demonstrate the well-established relationship between FU sensitivity and low TS expression (Beck *et al*, 1994; Peters *et al*, 2002), herein evaluated as enzymatic activity. In contrast, whether in the absence or presence of FA, FU sensitivity was not statistically associated with either 5′ or 3′ TS polymorphism (Figure 4). The absence of impact of 5′ TS genotype on FU sensibility closely agrees with our previous prospective clinical study (Etienne *et al*, 2002), reporting identical response rates between 2R/2R, 2R/3R and 3R/3R patients (genotype analysed on liver metastasis) in 88 patients receiving FU–FA chemotherapy. However, the present results are not in line with other recent clinical studies, most of which were conducted on rather small sets of colorectal cancer patients. Higher response rates were thus reported in 2R/2R patients by Pullarkat *et al* (2001) in 50 patients receiving protracted FU infusion and by Park *et al* (2002) in 24 capecitabine-treated patients. Also, lower downstaging was demonstrated in 3R/3R tumours by Villafranca *et al* (2001) on 66 rectal cancer patients receiving FU-based chemoradiotherapy protocols. A significant shorter survival rate in 3R/3R patients receiving FU-based adjuvant chemotherapy was demonstrated by Iacopetta *et al* (2001). The value of 5′ TS genotype for predicting fluoropyrimidine responsiveness and its use as a surrogate of TS measurement at the target level is still far from being clearly established, and requires additional large-scale prospective clinical studies including the recently reported G → C SNP within the triple tandem repeat (Kawakami and Watanabe, 2003; Mandola *et al*., 2003).

Methylenetetrahydrofolate reductase is a key enzyme of the folate metabolic pathway (Figure 1). Two SNPs (C677T and A1298C) associated with altered phenotypes have been described for this enzyme (Frosst *et al*, 1995; Weisberg *et al*, 1998). The mutated forms of these variants (i.e. 677TT and 1298CC) exhibit significantly lower enzymatic activity, and should theoretically lead to an accumulation of intracellular CH_2_FH_4_ concentrations as compared to wt forms. Consequently, C677T and A1298C MTHFR polymorphisms may influence the pharmacodynamics of fluoropyrimidines since they control the intracellular concentration of the specific reduced folate required for optimal TS inhibition. This hypothesis is supported by preclinical and clinical data (Danenberg and Danenberg, 1978; Houghton *et al*, 1981; Rustum *et al*, 1987; Keyomarsi and Moran, 1988; Chéradame *et al*, 1997a, 1997b) demonstrating the impact of CH_2_FH_4_ intratumoral concentrations on FU cytotoxicity. Recent clinical studies have suggested that MTHFR polymorphisms may be associated with methotrexate pharmacodynamics (Ulrich *et al*, 2001; Urano *et al*, 2002). However, no study has so far reported their impact on fluoropyrimidine sensitivity (Wisotzkey *et al*, 1999). First of all, the present data closely concord with the theoretical impact of C677T and A1298C polymorphisms on CH_2_FH_4_ intracellular pool: five out of six homozygous wt C677 variants and seven out of 10 homozygous wt A1298 variants exhibited nondetectable CH_2_FH_4_ concentrations, in contrast to six out of 13 T/T or C/T 677 variants, and four out of nine C/C or A/C 1298 variants (Figure 5, Tables 1 and 2). These data agree with those of Kawakami *et al* (2001a) who recently reported relationships between MTHFR genotypes and tumoral-reduced folates in gastrointestinal cancer .

The C677T genotype did not significantly influence FU cytotoxicity, whether in the absence or presence of optimal FA supplementation (Figure 6). However, FU sensitivity was related to A1298C MTHFR genotype: homozygous mutated cell lines (C/C) were the more sensitive, homozygous wt (A/A) were the more resistant, heterozygous cell lines (A/C) exhibiting intermediary sensitivity (Figure 7, Tables 1 and 2). The influence of A1298C MTHFR polymorphism on FU sensitivity was observed irrespective of the absence or presence of optimal FA supplementation. These stimulating results show a trend close to statistical significance (*P*=0.055 and 0.085 in the absence and presence of FA, respectively) and are thus consistent with the initial hypothesis that MTHFR genotypes associated with altered enzymatic activity may be more FU sensitive.

Altogether, it is hoped that the present data will encourage future studies to consider not only TS polymorphisms but also MTHFR polymorphisms as potential predictors of fluoropyrimidine responsiveness and/or toxicity. Both tumoral and constitutional genotype analyses should be taken into account in clinical prospective studies, particularly regarding the design of future FU-based chemotherapy study.
